# A Systematic Review of the Variability of Ventilation Defect Percent Generated From Hyperpolarized Noble Gas Pulmonary Magnetic Resonance Imaging

**DOI:** 10.1002/jmri.29746

**Published:** 2025-02-26

**Authors:** Vanessa M. Diamond, Laura C. Bell, Jeffrey N. Bone, Bastiaan Driehuys, Martha Menchaca, Giles Santyr, Sarah Svenningsen, Robert P. Thomen, Helen Marshall, Laurie J. Smith, Guilhem J. Collier, Jim M. Wild, Jason C. Woods, Sean B. Fain, Rachel L. Eddy, Jonathan H. Rayment

**Affiliations:** ^1^ BC Children's Hospital Research Institute University of British Columbia Vancouver British Columbia Canada; ^2^ Clinical Imaging Group Genentech San Francisco California USA; ^3^ Department of Radiology Duke University Durham North Carolina USA; ^4^ Department of Radiology University of Illinois at Chicago Chicago Illinois USA; ^5^ Department of Medical Biophysics University of Toronto Toronto Ontario Canada; ^6^ Translational Medicine Program The Hospital for Sick Children Toronto Ontario Canada; ^7^ Firestone Institute for Respiratory Health, the Research Institute of St. Joe's Hamilton McMaster University Hamilton Ontario Canada; ^8^ Department of Medicine McMaster University Hamilton Ontario Canada; ^9^ Department of Radiology, School of Medicine University of Missouri Columbia Missouri USA; ^10^ POLARIS, Section of Medical Imaging and Technologies, Division of Clinical Medicine, School of Medicine and Population Health University of Sheffield Sheffield UK; ^11^ Insigneo Institute University of Sheffield Sheffield UK; ^12^ Cincinnati Children's Hospital Medical Center University of Cincinnati Cincinnati Ohio USA; ^13^ Department of Pediatrics University of Cincinnati Cincinnati Ohio USA; ^14^ Department of Radiology University of Iowa Iowa City Iowa USA; ^15^ UBC Centre for Heart Lung Innovation University of British Columbia Vancouver British Columbia Canada; ^16^ Department of Radiology University of British Columbia Vancouver British Columbia Canada; ^17^ Department of Pediatrics University of British Columbia Vancouver British Columbia Canada

**Keywords:** hyperpolarized 129Xe, hyperpolarized 3He, hyperpolarized noble gas, non‐proton MRI, pulmonary MRI, ventilation defect percent

## Abstract

Hyperpolarized (HP) gas pulmonary MR ventilation images are typically quantified using ventilation defect percent (VDP); however, the test‐retest variability of VDP has not been systematically established in multi‐center trials. Herein, we perform a systematic review of the test‐retest literature on the variability of VDP, and similar metrics, generated from HP MRI. This review utilizes the Medline, EMBASE, and EBM Reviews databases and includes studies that assessed the variability of HP MRI VDP. The protocol was registered to PROSPERO: CRD42022328535. Imaging techniques and statistical analysis characteristics were extracted and used to group studies to evaluate the overall ability to pool data across grouped studies. The ability to pool data to provide systematic evidence was assessed using a modified COSMIN tool. A total of 22 studies with 37 distinct aims for repeated HP MRI acquisition or quantification were included. Studies were grouped into six categories based on HP gas and analysis type: repeated imaging (^129^Xe *n* = 13, ^3^He *n* = 12), interobserver repeated analysis (^129^Xe *n* = 4, ^3^He *n* = 4) or intraobserver repeated analysis (^129^Xe *n* = 1, ^3^He *n* = 2). Studies assessed variability using a variety of statistical tests including absolute difference, percent coefficient of variation, Bland‐Altman limits of agreement, coefficient of reproducibility, or the intra‐class correlation. Individual studies generally reported low variability of VDP (ICC range: 0.5–1.0; Bland‐Altman bias range: −6.9–20%), but there was an overall inability to pool data and provide a meta‐analysis due to methodological inconsistencies and small sample size. Overall, we found that VDP has low variability in most studies. However, inconsistent image acquisition and quantification methodologies between studies limits direct comparability and precludes grouping of study data for meta‐analyses. Despite early efforts to standardize HP MRI acquisition, further work is necessary to standardize VDP quantification to allow broader validation and clinical implementation.

**Evidence Level:** 2

**Technical Efficacy:** Stage 3

## 1 Introduction

Sufficient monitoring of lung health is necessary for disease management by respiratory care teams worldwide. Spirometry is routinely used to assess and monitor lung function, with forced expiratory volume in one second (FEV_1_) or forced vital capacity (FVC) being the primary clinical outcome measures [1, 2]. However, spirometry provides only a global measurement of total lung function that is known to be insensitive to disease heterogeneity and early small airways disease [2]. Chest x‐ray is commonly used to visualize and measure lung structure but is two‐dimensional and does not routinely provide functional information. Chest CT imaging provides three‐dimensional high spatial resolution and can indirectly provide functional information; however, radiation exposure limits frequent monitoring [3]. Consequently, there is a great need for tools that are safe and sensitive to early and heterogeneous alterations in lung function.

Hyperpolarized (HP) gas magnetic resonance imaging (MRI) of the lungs uses inhaled 3‐helium (^3^He) or 129‐xenon (^129^Xe) gas to measure lung function [4]. Results from these gases have been shown to not be directly comparable due to the nature of the different nuclei [5, 6, 7]; however, both have been established as safe and feasible in adults and pediatrics [8, 9, 10, 11]. The most common HP MRI technique utilizes the spin‐density of inhaled hyperpolarized nuclei to visualize and quantify the intrapulmonary distribution of the tracer gas (or “static ventilation”) and is now approved for clinical use in the United States and the United Kingdom [12, 13]. Ventilation defect percent (VDP) is a widely reported quantitative outcome measure derived from static ventilation MRI, which quantifies the fraction of the lungs that does not receive gas after a single inhalation during a breath hold maneuver. In many single‐center studies, VDP has been shown to be correlated with FEV_1_ across multiple pulmonary disorders [14, 15, 16]. Additionally, VDP has been shown to be highly sensitive to abnormal lung function and related to important patient outcomes across single‐center studies. For example, in chronic obstructive pulmonary disease (COPD), VDP demonstrated a significant increase over 2 years while FEV_1_ remained constant [17]. Another study showed that baseline VDP predicted future COPD exacerbations [18]. Compared to spirometry, VDP has also been shown to be more sensitive to abnormal lung function in people with cystic fibrosis (CF) and in children following hematopoietic stem cell transplantation [19, 20, 21, 22]. Additionally, in asthma, baseline VDP has been shown to be related to prior hospitalizations [23] and predictive of future exacerbations [24]. VDP has also been demonstrated to be highly sensitive to treatment response in COPD, CF, and asthma [7, 25, 26]. Taken together, these studies provide evidence for VDP as a powerful and clinically relevant pulmonary outcome measure. Some studies have endeavored to define thresholds for clinically important change in VDP to support clinical decisions [27, 28, 29, 30]. However, due to the nature of the relatively small and heterogeneous single‐center studies to date, unanswered questions remain surrounding the limits of normal and clinically important changes for VDP.

Of primary importance, the test–retest variability of VDP must be clearly established to determine limits of normal and clinically important changes and assess its utility in comparison to conventional measures of lung function. In this context, variability reflects how much an outcome can be expected to change over repeat assessments in a stable individual [31]; variability can result from multiple sources, including technological acquisition, inter‐ and intraobserver measurement variation, defect quantification technique, and bio‐physiologic variations, such as lung inflation state, that are unrelated to disease [32], as well as disease‐specific differences. An understanding of measurement variability allows clinicians and end‐users to better interpret clinical measurements; for example, the biological variability and measurement error of FEV_1_ is well established as 100 mL absolute change or 10% relative change, which underpins much of the interpretation of this test [33]. To date, the variability of VDP has not been well established beyond single center studies. Therefore, the purpose of this systematic review was to assess the current literature on the test–retest variability of VDP (and similar metrics) as an outcome measure of HP MRI across multiple sites and studies.

## 2 Materials and Methods

### 2.1 Search Strategy

The Medline, EMBASE, and EBM Reviews databases were searched by one author (V.M.D.) using the Ovid search platform. Key search terms included: “respiratory tract disease,” “magnetic resonance imaging,” “hyperpolarized noble gas,” “ventilation defect,” “reproducibility,” and “variability.” The full search queries are included in Supplement. Studies were included from database inception until October 20th, 2024, for each database. This systematic review and full electronic search strategy were registered with the international prospective register of systematic reviews (PROSPERO CRD42022328535) before beginning data extraction [34]. Gray literature (work published outside of traditional channels [35]) was not included in this review; only peer‐reviewed literature published in English was included. Two authors (V.M.D. and R.L.E.) independently screened all titles and abstracts, after which full‐text review for eligibility was performed independently on relevant titles and abstracts. A third author (J.H.R.) resolved conflicts in eligibility decisions.

### 2.2 Eligibility

For this review, we included publications that evaluated the variability of VDP in healthy people and those with clinically diagnosed respiratory disease through either repeated imaging or repeated measurement quantification. Studies will be grouped for comparison according to the type of variability assessed (i.e., repeated scans, and/or intra/interobserver repeated quantification). To be eligible, studies must have reported VDP as an outcome measure generated from HP MRI or a similar metric with previous terminology such as ventilated volume (VV) or reader defect volume. Studies must have also reported one or more of the following measures of statistical metrics for the reported outcome: absolute difference, percent coefficient of variation, Bland–Altman limits of agreement, coefficient of reproducibility, or the intra‐class correlation coefficient. Study design was not restricted. Duplicate publications and studies reporting duplicate data were excluded using the Covidence software [36]. Studies using animal models and studies assessing technology feasibility were excluded.

### 2.3 Quality Assessment

The study design and results were assessed using the COSMIN extended criteria for good reliability and measurement error by two authors (V.M.D. and R.L.E.) [37, 38]. Study outcomes were rated based on whether they assessed reliability or measurement error as defined by the Good Measurement Properties (GMP) guidelines [37, 38]. Reliability was defined as the proportion of variance in the measurements due to true differences between repeated scans and was rated sufficient if an intra‐class correlation (ICC) of greater than or equal to 0.7 was reported. If no ICC was reported, reliability was rated indeterminate. Measurement error was defined as systematic and random error in repeated VDP analysis measurements, that is not attributed to true changes in VDP (i.e., inter‐ or intra‐observer VDP measurement variability). COSMIN criteria define sufficient evidence of adequate measurement error if the smallest detectable change of limits of agreement is less than the minimal important change; as there is no consensus on the minimal important change (MIC) of VDP, all work assessing measurement error was thus rated indeterminate by these criteria.

The modified Grading of Recommendations Assessment, Development, and Evaluation (GRADE) system was used to assess the certainty of combined evidence across the included studies (Table S1) [38, 39, 40]. To accommodate study designs and the assessment of different aspects of variability, studies were grouped by the gas used (^3^He or ^129^Xe), then by the type of repeated outcome reported (repeated scans, and/or intra/interobserver repeated quantification), for a total of six groups. If studies reported multiple repeated outcome types (i.e., repeated scans and repeated analysis), the study appeared in multiple groups for each repetition type assessed. The following three factors were considered for the grade of the quality of evidence: inconsistency (heterogeneity of results and methods), imprecision (smaller sample sizes yield greater uncertainly and imprecision of results), and indirectness (repeatability assessed as primary aim). For the purposes of this study, COSMIN risk of bias was not evaluated because it focused on methodological reporting, rather than study design (i.e., acquisition or algorithmic methods, sample sizes, primary vs. secondary aim) and measurement repeatability results. Table S1 provides definitions for each factor and details regarding how downgrading was assessed. The GRADE certainty of combined evidence for each group begins as “high” and is downgraded to “moderate,” “low,” or “very low” if appropriate based on the three considered factors.

### 2.4 Data Extraction

All study characteristics and summary statistics were extracted by one author (V.M.D.). The following data were extracted using a standardized form: first author, year of publication, title, sample size, population description, MRI scanner make and model, MRI field strength, radiofrequency coil type, type of gas (i.e., ^3^He or ^129^Xe), polarization method and equipment, gas dosing, VDP (or equivalent outcome measure) quantification method, bias field correction approach, number of observers, time between scans, reported outcome measure(s), and reported statistical test(s): absolute difference, percent coefficient of variation, Bland–Altman limits of agreement, coefficient of reproducibility, or the intra‐class correlation coefficient. If studies reported multiple values for the collected data, all values were collected in this review. Extracted data are summarized in tabular form.

## 3 Results

**FIGURE 1:**
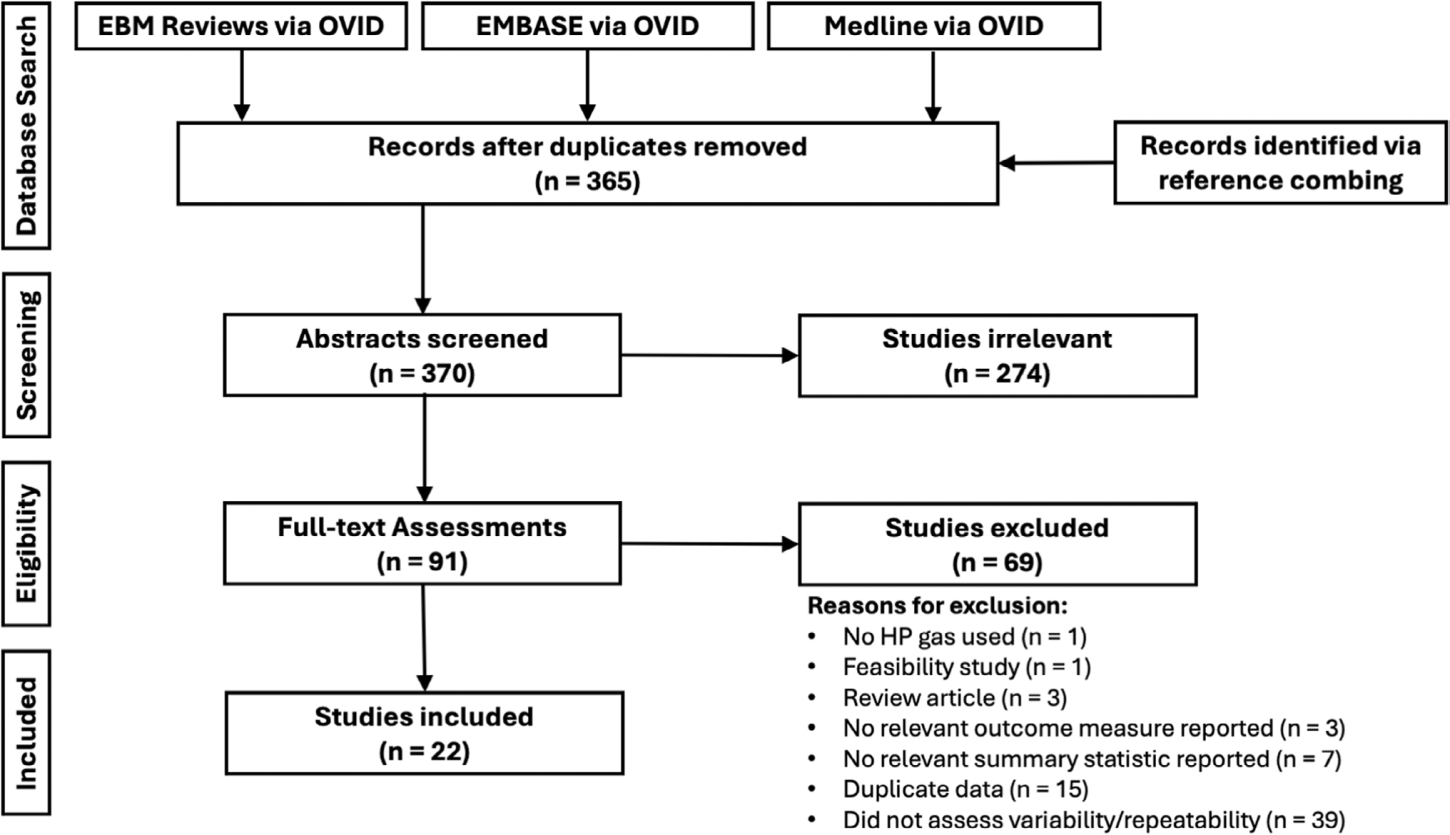
PRISMA flow diagram of database search results.

After identifying records and removing duplicates with the Covidence software, 379 records were identified (Figure 1). Abstracts were then screened for relevance, leaving 96 (26%) potentially relevant records with repeated HP MRI acquisition or quantification and 274 (74%) irrelevant records excluded. Full‐text assessment of 96 potentially relevant records was performed, leaving 22 (23%) eligible and 74 (77%) excluded records. Specific reasons for full‐text exclusions of studies are shown in Table S2. Table 1 provides a summary of the 22 included studies in alphabetical order with associated participant population, sample size, and MRI VDP (or similar measure) value. Table 2 provides a summary of the acquisition and quantification methodology, and Table 3 summarizes other elements of study design and statistical outcomes of the 22 included studies.

**TABLE 1:** Population characteristics and HP MRI outcome measures of included studies.

Study	Population	Sample size (*n*)	VDV/P	VV/%
1. Bashi et al. (2024)	PCD	7	3.68%^a^	NR
2. Couch et al. (2019)	HC	8	5.96%^a^	NR
	CF	18	15.96%^a^	
3. Diamond et al. (2023)	HC	4	NR	NR
	CF	12		
4. Ebner et al. (2017)	HC	10	5.6%^a^	NR
	Asthma	20	8.7%^a^	
5. Horn et al. (2014)	HC	13	NR	92.5%^a^
6. Hughes et al. (2018)	Horseshoe lung	1	NR	NR
	Asthma	1		
	COPD	4		
	Lung cancer	6		
7. Kirby et al. (2012)	CF, COPD and Asthma	15	0.92 L^a^	NR
8. Kirby et al. 2011)	CF	12	0.93 L^a^	NR
9. Marshall et al. (2021)	Asthma	33	NR	90.8%
10. Mathew et al. (2008)	HC	8	80 cm^3^ ^a^	NR
	COPD	16	70 cm^3^ ^a^	
11. Munidasa et al. (2023)	HC	7	2.65%^a^	NR
	CF	15	8.57%^a^	
12. Niles et al. (2013)	Asthma	13	252 mL^a^	5.37%^a^
13. Parraga et al. (2008)	HC	32	52 cm^3^ ^a^	NR
14. Roach, et al. (2022)	CF	37	9.20%^a^	NR
15. Smith, et al. (2020)	CF	29	9.5%^a^	NR
16. Stewart, et al. (2018)	HC	19		98.4%^a^
	COPD	5		71.4%^a^
	NSCLC	16		79.6%^a^
17. Svenningsen et al. (2021)	Asthma	7	NR	11%^a^
18. Svenningsen et al. (2014)	COPD	9	21%	NR
	Non‐CF Bronchiectasis		18%	
19. Walkup et al. (2024)	CF	38	5.0%^c^	NR
20. Woodhouse et al. (2009)	CF	5		NR
21. Zha et al. (2019)	CF	24	25.5%	NR
22. Zha et al. (2016)	EIB	6	1.11%^a^	NR

Abbreviations: CF = cystic fibrosis; COPD = chronic obstructive pulmonary disease; EIB = exercise‐induced bronchoconstriction; HC = healthy control; NR = not reported; NSCLC = nonsmall‐cell lung cancer; PCD = primary ciliary dyskinesia; VDV/P = average ventilation defect volume or percent (or equivalent metric); VV/% = average ventilated volume or percent (or equivalent metric).

^a^ Multiple value are reported in the study; the first reported value is included here.

**TABLE 2:** Study‐specific acquisition and quantification methodology.

Study	Vendor	Field strength	Sequence	Gas	Algorithm	Bias field correction
1. Bashi et al. 2024)	NR	NR	NR	Xe	Not stated	NR
2. Couch et al. (2019)	General Electric	1.5 T	2D	Xe	Linear binning	NR
	Philips	3 T	GE			
3. Diamond et al. 2023)	NR	NR	NR	Xe	K‐means clustering	NR
4. Ebner et al. (2017)	General Electric	1.5 T	3D GE	Xe	Linear Binning	NR
5. Horn et al. (2014)	General Electric	1.5 T	3D nGE	He	Threshold‐based region growing	NR
6. Hughes et al. (2018)	General Electric	1.5 T	3D	Xe	Thresholding & spatial fuzzy C‐means clustering	NR
			GE	He		
7. Kirby et al. (2012)	General Electric	3 T	2D GE	He	Manual & K‐means clustering	NR
8. Kirby et al. (2011)	General Electric	3 T	2D GE	He	K‐means clustering	NR
9. Marshall et al. (2021)	General Electric	1.5 T	2D GE	He	Threshold‐based region growing	No
10. Mathew et al. (2008)	General Electric	3 T	2D GE	He	Manual	No
11. Munidasa et al. (2023)	Siemens	3 T	2D GE	Xe	Mean‐anchored thresholding	N4ITK
12. Niles et al. (2013)	General Electric	1.5 T	2D	He	Manual Segmentation	NR
		3 T	GE			
13. Parraga et al. 2008)	General Electric	3 T	2D GE	He	Manual	NR
14. Roach et al. (2022)	General Electric Siemens Philips	3 T	2D GE	Xe	Manual & mean‐anchored thresholding	N4ITK & Linear RF
15. Smith et al. (2020)	General Electric	1.5 T	3D GE	Xe	Spatial fuzzy C‐means clustering	NR
16. Stewart et al. (2018)	General Electric	1.5 T	3D	Xe	Threshold‐based region growing	NR
			nGE	He		
17. Svenningsen et al. (2021)	General Electric	3 T	3D GE	Xe	K‐means clustering	NR
18. Svenningsen et al. (2014)	NR	NR	NR	He	Not stated	NR
19. Walkup et al. (2024)	General Electric	3T	2DGE	Xe	Manual & mean‐anchored thresholding	N4ITK
	Siemens					
	Philips					
20. Woodhouse et al. (2009)	Philips	1.5 T	3D GE	He	SNR‐anchored thresholding	NR
21. Zha et al. (2019)	General Electric	1.5 T	2D GE	He	K‐means clustering	N4ITK
22. Zha et al. (2016)	General Electric	1.5 T	2D GE	He	Manual & K‐means clustering	N4ITK

Abbreviations: GE = gradient echo; He = helium‐3; nGE = non‐gradient echo; NR = not reported; Xe = xenon‐129.

**TABLE 3:** Study design, reported statistics, and quality assessment for included studies.

Study	Repeat type	Scan repeat interval	Bland–Altman bias (LoA)	ICC	R or ME	GMP	
						R	ME
1. Bashi et al. (2024)	Scans	28 days	NR	0.47	R	−	NA
2. Couch et al. (2019)	Interobserver	NA	0.14 (−2.7, 3.0)	0.99	R	+	NA
3. Diamond et al. (2023)	Scans	20 min	0.22 (−3.06, 3.49)	0.48	Both	−	?
4. Ebner et al. (2017)	Scans	1 month	−0.04 (−3.19, 3.10)	0.60	Both	−	?
5. Horn et al. (2014)	Scans	10 min	−0.88 (−2.4, 0.64)	0.98	R	+	?
6. Hughes et al. (2018)	Scans—1 breath	≤ 10 min	0.72 (−3.0, 4.44)^d^	0.96	Both	+	?
7. Kirby et al. (2012)	Scans—2 breaths	≤ 10 min	0.20 (−6.14, 6.55)^d^	0.88	Both	−	?
8. Kirby et al. (2011)	Interobserver	NA	−0.9 (−20.0, 18.2) ^c^	0.58^c^	Both	+	?
9. Marshall et al. (2021)	Interobserver	NA	−1.1 (−6.7, 4.5) ^c^	0.85^c^	Both	+	?
10. Mathew et al. (2008)	Intraobserver	NA	NR	0.98^c^	Both	+	?
11. Munidasa et al. (2023)	Interobserver	NA	NR	0.96^c^	Both	+	?
12. Niles et al. (2013)	Scans	7 days	−3 (−11, 5)	NR	ME	NA	?
13. Parraga et al. 2008)	Intraobserver	NA	NR	NR	ME	NA	?
14. Roach et al. (2022)	Scans	5 mins	0.12 (−1.86, 2.1)	1.00	Both	+	?
15. Smith et al. (2020)	Scans	7 mins	NR	0.96	R	+	NA
16. Stewart et al. (2018)	Scans	7 days	NR	0.98	R	−	NA
17. Svenningsen et al. (2021)	Scans	Same‐day^a^	−0.1 (−4.12, 3.91)	0.93	Both	+	?
18. Svenningsen et al. (2014)	Scans	1 month	−1.25 (−8.80, 6.31)	0.68	Both	−	?
19. Walkup et al. (2024)	Scans	7 days	NR	0.89^c^	R	+	NA
20. Woodhouse et al. (2009)	Interobserver	NA	2.91 (−4.52, 10.30)	0.91^c^	R	+	NA
21. Zha et al. (2019)	Scans	7 mins	NR	NR	ME	NA	?
1. Bashi et al. (2024)	Scans	7 days	NR	NR	ME	NA	?
	Interobserver	NA	NR	NR	ME	NA	?
2. Couch et al. (2019)	Scans	8 h	0.5 (−3.85, 4.35)^c^	NR	ME	NA	?
3. Diamond et al. (2023)	Scans	15 mins	0.2 (−1.4, 1.8)	0.99	Both	+	?
4. Ebner et al. (2017)	Scans	16 months	0.8 (−6.9, 8.5)^c^	0.97	Both	+	?
5. Horn et al. (2014)	Scans	Multi^b^	NR	0.54	Both	−	?
6. Hughes et al. (2018)	Scans	Multi^b^	NR	0.46	Both	−	?
7. Kirby et al. (2012)	Scans	24 h	−3 (−14, 8)^c^	NR	Both	+	?
8. Kirby et al. (2011)	Interobserver	NA	0 (−4, 3)^c^	0.97^c^	Both	+	?
9. Marshall et al. (2021)	Intraobserver	NA	0 (−3, 2)^c^	0.99^c^	Both	+	?
10. Mathew et al. (2008)	Scans	3 weeks	NR	0.61^c^	R	+	NA
11. Munidasa et al. (2023)	Scans	36 mins	0.12 (−3.2, 3.4)	NR	ME	NA	?
12. Niles et al. (2013)	Scans	1 month	NR	NR	NA	NA	NA
13. Parraga et al. 2008)	Scans	30 mins	−3.7 (−7.7, 0.15)	NR	ME	NA	?
14. Roach et al. (2022)	Scans	1–2 weeks	2.25 (−6.04, 10.54)	0.95	Both	+	?
15. Smith et al. (2020)	Interobserver	NA	0.22 (LoA NR)	NR	ME	NA	?

Abbreviations: GMP = good measurement properties; GMP: sufficient (+); insufficient (−); indeterminate (?); not applicable (NA); ICC = intraclass correlation coefficient; LoA = limits of agreement; ME = measurement error; R = reliability.

^a^ Time difference not specified.

^b^ Twice on day 1, once on day 2, and once 2 weeks post day 1.

^c^ Multiple values reported in the study, the first reported value is included here (except study 5 where the first reported value from the semi‐automated approach is included here).

^d^ Figure reported in the paper, values acquired by personal communication with authors.

### 3.1 Participants

The included studies explored nine different participant populations: CF [6, 41, 42, 43, 44, 45, 46, 47, 48, 49], COPD [5, 6, 50, 51, 52], asthma [6, 50, 53, 54, 55, 56], lung cancer [5, 50], bronchiectasis [52], exercise‐induced bronchoconstriction [57], horseshoe lung [50], primary ciliary dyskinesia [58], and healthy people [5, 41, 43, 48, 51, 53, 59, 60] (Table 4). Individual study sample sizes ranged from 6 to 40 participants, with 13 studies evaluating exclusively adult populations [5, 6, 42, 50, 51, 52, 53, 54, 55, 56, 57, 59, 60], seven pediatric populations—defined as less than 18 years of age [41, 43, 45, 46, 48, 49, 58], and two in combination [44, 47].

**TABLE 4:** Study population and design.

Study design element	Number of studies
Total included studies	22
Population	
Disease group	
Healthy	8 (36%)
Asthma	6 (27%)
Bronchiectasis	1 (5%)
Chronic obstructive pulmonary disease	5 (23%)
Cystic fibrosis	10 (45%)
Exercise‐induced bronchoconstriction	1 (5%)
Horseshoe lung	1 (5%)
Lung cancer	2 (9%)
Primary ciliary dyskinesia	1 (5%)
Adult	13 (59%)
Pediatric	7 (32%)
Adult and pediatric	2 (9%)
Number of sites	
1 Site	17 (77%)
2 Sites	3 (14%)
4 Sites	2 (9%)
Scans	
Observers	
Single observer	3 (14%)
Multiple observers	7 (32%)
Not reported	14 (64%)
Same‐day repeat	13 (59%)
Repeat visit	13 (59%)
Short‐term ≤ 1 month	12 (45%)
Long‐term ≥ 1 month	1 (5%)
Single scan—Repeat quantification	8 (36%)

### 3.2 Design

The included studies employed a variety of study designs related to the number of sites for data collection, repeat type, and where applicable, repeat interval. Seventeen studies collected data at a single site [5, 6, 42, 43, 44, 46, 47, 48, 50, 51, 52, 53, 54, 57, 58, 59, 60], three studies combined data from two sites [41, 55, 56], and two studies from four sites [45, 49]. Eight studies performed a single scan and compared multiple quantifications [6, 41, 42, 50, 55, 56, 57, 60], of which three compared quantifications by the same observer [6, 42, 56], and seven between multiple observers [6, 41, 50, 55, 56, 57, 60]. Thirteen studies performed same‐day repeated scans [5, 43, 44, 45, 46, 48, 49, 51, 53, 54, 56, 59, 60], and 13 performed repeated scans separated by more than 1 day [5, 42, 43, 44, 47, 48, 49, 51, 52, 55, 56, 58, 60]. For those separated by ≥ 1 day, the median time between them ranged from 1 day to 16 months.

### 3.3 Image Acquisition

The included studies reported three different MRI vendors: General Electric [5, 6, 41, 42, 44, 45, 47, 49, 50, 51, 53, 54, 55, 56, 57, 59, 60], Siemens [43, 45, 49], and Philips [41, 45, 46, 49]. Three studies reported a combination of vendors [41, 45, 49], and three studies did not report vendors [48, 52, 58] (Table 5). There were nine studies that reported 1.5 T field strength [5, 44, 46, 47, 50, 53, 54, 57, 59], eight using 3 T [6, 42, 43, 45, 49, 51, 56, 60], two using a combination [41, 55] and three that did not report field strength [48, 52, 58]. For hyperpolarized gas imaging acquisition, 12 studies used 2D multi‐slice sequences [6, 29, 41, 42, 45, 47, 49, 51, 54, 55, 57, 60] and seven used 3D sequences [5, 44, 46, 50, 53, 56, 59]; three did not report sequence details [48, 52, 58]. Seventeen used gradient echo sequences [6, 41, 42, 43, 44, 45, 46, 47, 49, 50, 51, 53, 54, 55, 56, 57, 60], and two used a non‐gradient echo sequence [5, 59]. For specialized HP MRI hardware, twelve used commercially built radiofrequency coils [5, 6, 42, 43, 44, 49, 50, 51, 53, 54, 59, 60], two used home‐built coils [46, 56], two used a combination [41, 55], and six did not report [45, 47, 48, 52, 57, 58]. Of these, eight studies used flexible coils [5, 43, 44, 49, 50, 53, 54, 59], six used rigid coils [6, 42, 46, 51, 56, 60], and two used a combination of coil types [41, 55].

**TABLE 5:** Image acquisition methods.

Image acquisition element	Number of studies
MRI Specs	
General Electric	17
Signa HDx	9
Excite	5
Discovery	2
Siemens	3
Magnetom prismafit	3
Philips	4
Eclipse	1
Achieva	2
Field strength	
1.5 Tesla	9
3 Tesla	8
1.5 and 3 Tesla	2
Not reported	3
Sequence	
Excitation scheme	
2D	12
3D	7
Not reported	3
Gradient echo	16
Non‐gradient echo	2
No sequence reported	3
Coil specs	
Type	
Flexible	8
Rigid	6
Manufacturer	
Home built	2
Commercial	12
Multiple coils	2
Not reported	6

### 3.4 Gas

The included studies used different hyperpolarized gases and techniques for dosing and administration. Eleven of the studies utilized ^3^He [6, 42, 46, 47, 51, 52, 54, 55, 57, 59, 60], nine used ^129^Xe [41, 43, 44, 45, 48, 49, 53, 56, 58], and two used a combination of both gases [5, 50] (Table 6). Of the studies using ^129^Xe, four used an enriched blend [41, 44, 53, 56], five did not specify [5, 45, 48, 49, 50, 58], and one used multiple gas blends [43]. Twelve studies used commercially built gas polarizers [6, 41, 42, 43, 46, 47, 51, 55, 56, 57, 59, 60], one used a home‐built polarizer [44] and two used multiple types [5, 50]. Gas was produced at a range of different volumes and administered at different initial lung inflation volumes. Seven studies used a standard mixture of HP gas and buffer gas for all participants [5, 41, 50, 53, 54, 56, 59], nine studies tailored the ratio of HP gas to buffer gas and total bag volume to the participant [6, 42, 43, 44, 46, 49, 51, 55, 60], five used multiple approaches [41, 46, 49, 50, 56], and seven studies did not report gas ratios [5, 45, 47, 48, 52, 57, 58]. A total of 15 distinct methods were reported for gas dosing and administration across the 22 studies.

**TABLE 6:** Hyperpolarized gas characteristics, equipment, and dosing.

Characteristics	Number of Studies
Gas	
Helium‐3	11
Xenon‐129	9
Unspecified	6
Enriched	4
Multiple blends	1
Combination of gasses	2
Polarizer	
Commercial	12
Home built	1
Multiple	2
Not reported	6
Total gas dosing volume	
1 L	13
1/6 TLC	2
0.4–1 L (height dependent)	1
Not reported	7
Initial lung volume	
Functional residual capacity	15
Multiple methods	1
Not reported	7
Dosing method	
Total different approaches	15
Participant dependent dosing	9
Standard dosing for all participants	7
Multiple methods	5
Not reported	7

### 3.5 Image Quantification

There were two main categories of image quantification methods: semiautomated segmentation [5, 6, 41, 42, 43, 44, 45, 46, 47, 48, 49, 50, 53, 54, 56, 57, 59] and manual segmentation [6, 45, 49, 51, 55, 57, 60]; two studies did not report their approach [52, 58] (Table 7). Five studies used bias field corrections to mitigate radiofrequency inhomogeneity [43, 45, 47, 49, 57], two explicitly reported not using any bias field correction [51, 54], and the rest did not report their bias field correction approach. Of the four studies that used bias field corrections, all four used an N4ITK correction, and one of these compared a linear RF correction to an N4ITK correction [45]. The semiautomated methods included various underlying algorithms to define ventilation versus ventilation defect within the lungs, including linear binning [41, 53], clustering including k‐means and fuzzy c‐means [6, 42, 44, 47, 48, 50, 56, 57], mean‐anchored thresholding [43, 45, 61], threshold‐based region growing [5, 54, 59] and other thresholding methods [46, 50]. From these various methods, six different terms for similar metrics that quantify defect were reported: ventilation volume (VV) [6, 46, 50, 54], ventilation volume percent (VV%) [5, 46, 54, 55, 59], ventilation defect volume whole‐lung (VDV) [6, 42, 55] or single slice [51, 60], ventilation defect percent whole‐lung (VDP) [41, 42, 43, 44, 45, 47, 48, 49, 52, 53, 56, 57, 58] or single slice [43], and reader defect volume [49].

**TABLE 7:** Image quantification and statistical analysis methods.

Quantification and/or analysis specification	Number of studies
Pipeline	
Semiautomated	17
Manual	7
Not reported	2
Bias field correction	
Used	4
Not used	3
Not reported	15
Underlying algorithm	
Linear binning	2
K‐means or fuzzy c‐means clustering	8
Mean‐anchored thresholding	3
Other thresholding	5
Outcome measure	
Ventilation volume (VV)	4
Ventilation volume percent, (VV%)	5
Ventilation defect volume (VDV)	3
Centre slice only	2
Ventilation defect percent (VDP)	13
Single slice only	1
Reader defect volume	1
Statistical test	
Absolute difference	2
Coefficient of variation	7
Bland Altman	15
Limits of agreement	14
Coefficient of reproducibility	1
Intra‐class correlation coefficient	16

### 3.6 Reliability and Measurement Error

**FIGURE 2:**
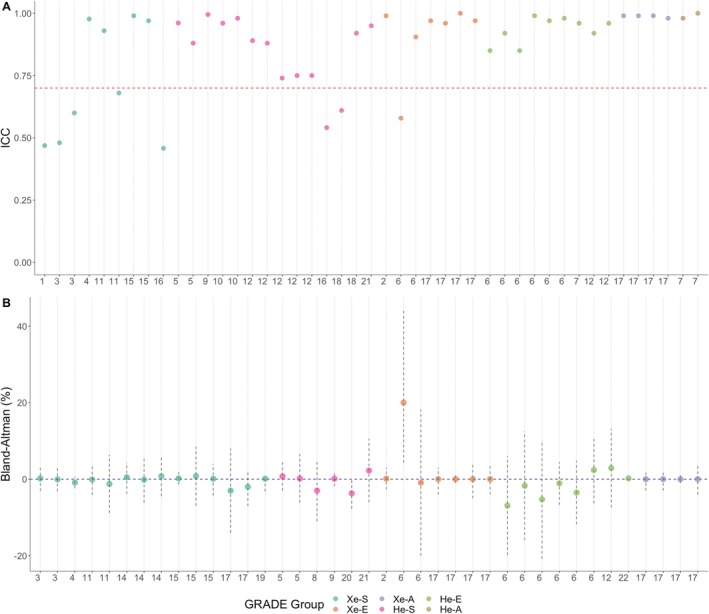
Plot presenting reported outcomes for each aim from each included study numbered as in Table 1. ICC (A) and Bland–Altman Bias (points) and Limits of Agreement (dashed vertical lines) (B) from each study reporting VDP or VV%. In panel A, the red dashed line represents an acceptable ICC (0.70). In panel B, the navy dashed line represents no bias. High reliability and low variability of VDP were found in individual studies. Xe‐S = Xe repeated scan; Xe‐E = Xe interobserver repeat; Xe‐A = Xe intraobserver repeat; He‐S = He repeated scan; He‐E = He interobserver repeat; He‐A = He intraobserver repeat.

Variability of VDP and equivalent metrics was assessed using absolute difference [43, 54], coefficient of variation [5, 6, 42, 43, 54, 59, 60], Bland–Altman bias (mean difference) [41, 42, 43, 44, 45, 46, 47, 48, 49, 50, 53, 54, 55, 56, 57], limits of agreement [41, 42, 43, 44, 45, 46, 47, 48, 49, 50, 53, 54, 55, 56], coefficient of reproducibility [43], and intraclass correlation [5, 6, 41, 43, 44, 47, 48, 50, 51, 52, 53, 54, 55, 56, 58, 59] (Table 7). Reported findings for the ICC and Bland–Altman analysis are presented in Figure 2. ICC ranged from 0.46 to 1.0, with six studies with ICC < 0.70. Bland–Altman bias range ranged from −6.9% to 20%, with the majority of studies near zero bias. We note that the one study that reported the 20% bias was using a basic thresholding method, against which an improved, semi‐automated fuzzy c‐means method was compared showing a bias of −0.9% (see two side‐by‐side studies labeled “6” in Figure 2B) [50]. Fifteen of the studies assessed the reliability of VDP and similar metrics [5, 6, 41, 43, 44, 47, 48, 50, 51, 52, 53, 54, 55, 56, 58] and 16 studies assessed measurement error of VDP and similar metrics [5, 6, 42, 43, 44, 45, 46, 47, 48, 49, 50, 54, 56, 57, 59, 60].

### 3.7 Good Measurement Properties (GMP) and GRADE Summary

The GMP ratings are shown in Table 8, along with a summary of the study repeat type, repeat interval, and reported statistical metrics. For the GMP analysis, aims were rated based on whether they assessed reliability (*n* = 8), measurement error (*n* = 9), or both (*n* = 19). Of the 27 aims that assessed reliability, 19 were rated Sufficient (ICC ≥ 0.70), and eight were rated Insufficient (ICC < 0.70) based on the GMP guidelines. All 29 aims assessing Measurement Error were rated as indeterminate as there is no consensus on MIC for VDP to be used in the GMP analysis.

**TABLE 8:** GRADE for all groups of studies.

Group		Studies, no. of aims	Factor	Rating	Grade
^129^Xe	Repeated scans	(Bashi, 2024), 1	Inconsistency	Very serious	Very low
		(Diamond, 2023), 2	Imprecision	Acceptable	
		(Ebner, 2017), 1	Indirectness	Acceptable	
		(Munidasa, 2023), 2			
		(Niles, 2013), 1			
		(Roach, 2022), 1			
		(Smith, 2020), 2			
		(Stewart, 2018), 1			
		(Svenningsen, 2021), 1			
		(Walkup, 2024), 2			
	Interobserver repeat	(Couch, 2019), 1	Inconsistency	Very serious	Very low
		(Hughes, 2017), 1	Imprecision	Serious	
		(Niles, 2013), 1	Indirectness	Acceptable	
		(Svenningsen, 2021), 1			
	Intraobserver repeat	(Svenningsen, 2021), 1	Inconsistency	Acceptable	Very low
			Imprecision	Very Serious	
			Indirectness	Acceptable	
^3^He	Repeated scans	(Horn, 2014), 2	Inconsistency	Very serious	Very low
		(Kirby, 2011), 1	Imprecision	Acceptable	
		(Marshall, 2021), 1	Indirectness	Serious	
		(Matthew, 2008), 2			
		(Parraga, 2008), 2			
		(Stewart, 2018), 1			
		(Svenningsen, 2014), 1			
		(Woodhouse, 2009), 1			
		(Zha, 2019), 1			
	Interobserver repeat	(Hughes, 2017), 1	Inconsistency	Very serious	Very low
		(Kirby, 2012), 1	Imprecision	Very serious	
		(Parraga, 2008), 1	Indirectness	Acceptable	
		(Zha, 2016), 1			
	Intraobserver repeat	(Kirby, 2012), 1	Inconsistency	Very serious	Very low
		(Kirby, 2011), 1	Imprecision	Very serious	
			Indirectness	Acceptable	

Study aims were categorized into six different groups for the GRADE analysis based on the distinct aim explored, with each group receiving a “Very Low” rating (Table 8). “Very Low” ratings were driven by different image acquisition and VDP quantification methods in five groups (i.e., very serious inconsistency), small sample sizes in four groups (i.e., serious or very serious imprecision), or because variability was not assessed as a primary aim (i.e., serious indirectness). Full details for inconsistency, imprecision, and indirectness are shown in Table 8, with definitions of all criteria in Table S1. The quantitative results in Figure 2 are presented by GRADE group using different colors.

## 4 Discussion

VDP, derived from HP MRI, is a feasible and sensitive measure of lung function with applicability across a spectrum of pulmonary disorders [62]. Additionally, there has been recent FDA approval for the use of hyperpolarized 129‐xenon gas as a contrast agent for use with pulmonary MRI in those 12 years of age and older; however, approval of a quantification methodology and of VDP as an outcome measure is yet to occur [63]. Further, in the United Kingdom, the Medicines and Healthcare products Regulatory Agency (MHRA) and Good Manufacturing Process (GMP) have authorized the Xenon Polariser Laboratory at the University of Sheffield to use hyperpolarised 129‐xenon gas for human use and use in clinical trials [64]. To continue to progress in clinical implementation, the variability of VDP must be well understood. Thus, in this systematic review, we summarized the current literature regarding the variability of VDP. Overall, there was high reliability and low test–retest, intraobserver, and interobserver variability of VDP reported within individual studies, as outlined in Figure 2. The main result of our study is that, despite studies from single centers demonstrating low variability of VDP, the inconsistent methodological approaches in image acquisition, post‐processing techniques, and statistical analyses between studies precluded a meta‐analysis of pooled variability data and a consequent very low GRADE certainty of combined evidence. Additionally, the lack of consensus regarding the MIC for VDP prevented the assessment of measurement error. This study highlights the importance of ongoing efforts to standardize VDP quantification to allow for multi‐center interoperability as well as meaningful clinical interpretation of quantitative HP MRI.

The main methodological differences between the studies that preclude direct comparison can be grouped in three distinct categories: (1) disease under study, (2) image acquisition protocols, and (3) image quantification methodologies. First, the included studies evaluated seven different lung conditions, and not all studies included healthy participants for comparison. Given the unique manifestations of different lung diseases, whether there is disease‐specific physiologic variability in VDP is not well understood. Disease‐specific variability of spirometric outcomes has not been well established either; however, attempts have been made to define this in CF, COPD, and interstitial lung disease [65, 66, 67, 68]. It has been shown that only a small amount (2%–4%) of spirometric variability can be explained by patient characteristics such as age, sex, height, smoking status, and FEV_1_ [69]. Taking disease‐specific variability into consideration may help to explain some of the remaining variability in spirometric outcomes and should be considered when reporting the variability of VDP. Disease‐specific differences have been considered in single‐center investigations of VDP in people with CF [42], COPD [6], and asthma [6, 27, 28], each reporting a disease‐specific MIC. The majority of these studies have actually recommended that the clinically relevant threshold for VDP should be ~2% [27, 28, 45]; however, this value requires validation in larger and broader patient cohorts for further confidence in interpretation.

Second, there were methodological differences between the included studies with respect to MRI hardware, pulse sequences, and gas dosing and administration procedures. Recommendations for image acquisition protocols across the major MRI vendors have now been published [12]; however, only three of the 21 included studies were conducted after these recommendations were published, and head‐to‐head cross‐platform comparisons have yet to be performed. Reassuringly, however, studies comparing VDP between 2D versus 3D pulse sequences [70, 71] and gradient echo versus balanced steady‐state free precession sequences [72] have shown these technical factors to have minimal impact on VDP. Furthermore, studies included in this review included 15 different hyperpolarized gas dosing strategies, included both ^3^He and ^129^Xe, and inconsistently reported gas polarization or dose equivalent volume. Studies directly comparing the use of different inhaled gases show that they are not comparable, as ^3^He VDP is consistently lower than that of ^129^Xe [5, 6, 7]. Further, systematic differences in lung inflation can bias results, with lower lung inflation volumes shown to result in higher VDP [32]. Additionally, the methodology for obtaining the anatomical images taken with traditional proton MRI techniques may impact the calculated VDP. Though no studies have directly assessed the impacts of moving participants and switching from the specialized ^129^Xe chest coil to a ^1^H coil versus using the embedded body coil without moving the participants, a study explored the impacts of obtaining the anatomical image during the same breath‐hold as the ^129^Xe image and during a second volume‐matched breath hold found significant differences in the resulting VDP [50]. Finally, the dose equivalent volume (DEV) of the inhaled HP gas (which is the product of the isotopic fraction, nuclear spin polarization, and the total volume of the inhaled HP gas) is related directly to the observed signal‐to‐noise ratio (SNR) in HP MRI ventilation imaging [12, 71] and is inconsistently reported in the included studies. The impact of SNR on variability has not been thoroughly assessed, but it has been suggested that a minimum SNR threshold must be met to ensure consistent VDP quantification [71, 73] and its impact on outcome variability has not been robustly assessed. Further efforts to standardize recommendations around lung inflation volume, DEV, and SNR, similar to work done on acquisition protocols [12], are necessary next steps towards inter‐institutional standardization and better understanding of these factors on the variability of VDP.

Third, in the reviewed studies, there was a very wide variety of image analysis pipelines for the segmentation of images and the definition of defect, which could have a significant impact on the variability of VDP. The major differences in the pipelines include the use of manual or semi‐automated approaches, the nature of the underlying quantification algorithm, and the definition of “defect.” It is also important to note that these sources of variability are also present in the assessment of lung volume from anatomical scans used in conjunction with ventilation scans to assess “defect” in these methodologies. Over time, novel approaches have been developed and currently there is no clear “gold standard” for the definition of VDP. Current manual approaches require highly trained personnel and are subject to observer bias. Semi‐automated methods have been shown to be less variable than manual methods [6, 50], though they are still subject to observer bias and bias introduced by varying underlying algorithms and definitions of ventilation defect. We also note that studies which conducted repeated scans versus repeated analyses (inter‐ or intra‐observer) answer different fundamental questions about VDP repeatability, and so we grouped these separately for evaluation of combined evidence.

The impacts of using specific clustering and thresholding methods to define VDP have been explored elsewhere and underscore the inappropriateness of directly comparing or combining VDP derived using different pipelines. For instance, a comparison of VDP from linear binning and adaptive k‐mean clustering highlights how each classifies signals differently and ultimately, classifies different defect volumes [73]. In a separate comparison of adaptive thresholding and k‐means clustering, VDP was consistently higher with adaptive thresholding [74]. A threshold may be selected based on how well it discriminates between health and disease, and adjusting that threshold impacts what the algorithm defines as defect [20, 75]. Work exploring five different VDP quantification methods, including variations of linear binning and thresholding, found differences in the abilities for each method to distinguish health from disease that vary for different disease groups [76]. Additionally, some studies used bias field corrections to correct radiofrequency field inhomogeneities when using flexible vest coils [77]; however, the specific implications of using different bias‐field correction tools on VDP are not well established, and there is no consensus on which bias‐field correction is superior (or indeed, whether it is necessary at all). Future automated algorithms and/or deep learning models to quantify VDP have the potential to further eliminate variability from observers using manual or semi‐automated approaches, thus improving the overall reliability of outcome measures, but no studies using these tools were included in this review. Further, there is a lack of literature exploring the impacts of the above‐mentioned variables (i.e., algorithm used, bias field corrections, etc.) on the variability of the anatomical scans used to assess total lung volume. These variables in the assessment of total lung volume will directly impact the repeatability of VDP outcomes. Finally, some studies assessed the intra‐observer variability of their quantification process, while others assessed inter‐observer variability. However, not all studies reported whether VDP assessments were computed by one or many observers. Together, these differences across study designs limited the overall ability to pool data to provide systematic evidence and precluded the direct comparison of results between studies.

### 4.1 Limitations

The primary limitation of this study is the potential for missed evidence. Literature may have been missed if it was not identified using our published search strategy (PROSPERO CRD42022328535) in one of the selected databases, reducing the number of possible studies included in the review. To mitigate this risk, the search strategy was bolstered by the manual addition of potentially eligible studies by all members of the authorship group, who are experts in the field. Gray literature was not included in this review, which may have included additional data not captured with our search strategy. Another limitation to note is that each of the 22 included studies was conducted at one of seven different sites, which could impact the generalizability of the results. Finally, the findings of this review are weakened by the inability to confidently group studies and perform a meta‐analysis on the pooled results. We acknowledge that the COSMIN tool, though validated and standardized, may not capture all nuances associated with VDP measurement from HP MRI that is still an emerging field; thus, we used a modified version to focus only on GMP and GRADE, excluding the risk of bias tool. The COSMIN risk of bias checklist focuses on methodological reporting, making it less relevant to the objectives of this study. This modified tool allowed the results to focus primarily on study design and VDP measurement variability metrics.

### 4.2 Conclusion

The reported variability of VDP is generally low in most individual studies, supporting its use as an imaging pulmonary outcome measure. However, direct comparison and aggregation of variability data across studies is not possible, primarily due to inconsistencies in study design and VDP quantification approaches. These study design inconsistencies, rather than fundamental flaws in the studies themselves, are what lead to the overall “very low” certainty of combined evidence reported in this systematic review. While the individual study results are reassuring, especially in the context of implementation of single‐center longitudinal monitoring of disease progression and treatment response, this review has highlighted a clear need in the field to establish a standard VDP quantification methodology. This is especially relevant if this technique is to be used to aggregate or compare data between centers for clinical trial or registry purposes. Efforts towards standardizing quantification methods can be made through large‐scale data registry projects that assess different methods and provide recommendations for standardization. This standardization effort is crucial to the advancement of HP MRI into clinical practice and clinical trials.

## Supporting information


**Data S1.** Supporting Information.
