# Supplementary material for: A Systematic Review of the Variability of Ventilation Defect Percent Generated From Hyperpolarized Noble Gas Pulmonary Magnetic Resonance Imaging
Source: J Magn Reson Imaging. 2025 Feb 26;62(3):625–39. doi: 10.1002/jmri.29746 (PMC12335342; doi:10.1002/jmri.29746)
Supplement: Supplementary file 1 — Data S1. Supporting Information. [file JMRI-62-625-s001.docx]

**Appendices**

**Online Supplement**

**Supplement**

Search Queries – PROSPERO Registration ID: CRD42022328535 <https://www.crd.york.ac.uk/PROSPEROFILES/328535_STRATEGY_20220606.pdf>

Ovid EBM Reviews; All EBM Reviews

1 (resp* tract diseas* or resp* diseas* or copd or chronic obstructive pulmonary diseas* or asthma* or obstructive lung diseas* or obstructive pulmonary diseas* or cystic fibrosis or cf or (lung adj3 disease*) or healthy or healthy control).mp.

2 ((magnetic resonance adj2 imag*) or MRI or magnetic resonance imag* or mr or pulmonary mri* or lung mri* or vent* magnetic resonance imag*).mp.

3 1 and 2

4 (((hyperpolarized 129Xe* or hp 129Xe* or hyperpolarized 129-Xe* or hp 129-Xe* or hyperpolarized 129 Xe* or hp 129 Xe* or (hyperpolarized adj3 Xe*) or (hp adj3 Xe*) or Xe-129 or Xenon-129 or (hyperpolarized adj3 Xe-129) or (hyperpolarized adj3 Xenon-129) or 129Xe* or (hyperpolarized adj3 He*) or (hp adj3 He*) or 129Xe*) adj3 MRI) or (129Xe* adj3 magnetic resonance imag*) or (129-Xe* adj3 MRI) or (Xe* adj3 MRI) or (129-Xe* adj3 magnetic resonance imag*) or (Xe* adj3 magnetic resonance imaging*) or 129xe-mri or (He* adj3 MRI) or (He* adj3 magnetic resonance imag*) or (hyperpolarized adj3 gas*) or (hyperpolarized adj3 noble gas*)).mp.

5 3 and 4

6 (ventilation defect percent* or vdp or ventilation defect* or ventilation volume percent* or (ventilation adj3 percent*) or ventilated volume percent* or (ventilated adj3 percent*) or (vent* adj3 volume) or ventilation heterogeneity index or vhi or (ventilat* adj3 hetero*)).mp.

7 5 and 6

8 (variab* or reproducib* or (test adj3 test) or test re-test or repeat* or replic*).mp.

9 7 and 8

Ovid EMBASE Embase

1 exp respiratory tract disease/

2 (resp* tract diseas* or resp* diseas* or copd or chronic obstructive pulmonary diseas*

or asthma* or obstructive lung diseas* or obstructive pulmonary diseas* or cystic fibrosis or cf or (lung adj3 disease*) or helahty or healthy control).mp.

3 1 or 2

4 exp nuclear magnetic resonance imaging/

5 ((magnetic resonance adj2 imag*) or MRI or magnetic resonance imag* or mr or pulmonary mri* or lung mri* or vent* magnetic resonance imag*).mp.

6 4 or 5

7 3 and 6

8 exp xenon/ or exp helium/

9 (((hyperpolarized 129Xe* or hp 129Xe* or hyperpolarized 129-Xe* or hp 129-Xe* or hyperpolarized 129 Xe* or hp 129 Xe* or (hyperpolarized adj3 Xe*) or (hp adj3 Xe*) or Xe-129 or Xenon-129 or (hyperpolarized adj3 Xe-129) or (hyperpolarized adj3 Xenon-129) or 129Xe* or (hyperpolarized adj3 He*) or (hp adj3 He*) or 129Xe*) adj3 MRI) or (129Xe* adj3 magnetic resonance imag*) or (129-Xe* adj3 MRI) or (Xe* adj3 MRI) or (129-Xe* adj3 magnetic resonance imag*) or (Xe* adj3 magnetic resonance imaging*) or 129xe-mri or (He* adj3 MRI) or (He* adj3 magnetic resonance imag*) or (hyperpolarized adj3 gas*) or (hyperpolarized adj3 noble gas*)).mp.

10 8 or 9

11 7 and 10

12 (ventilation defect percent* or vdp or ventilation defect* or ventilation volume percent* or (ventilation adj3 percent*) or ventilated volume percent* or (ventilated adj3 percent*) or (vent* adj3 volume) or ventilation heterogeneity index or vhi or (ventilat* adj3 hetero*)).mp.

13 11 and 12

14 exp reproducibility/

15 (variab* or reproducib* or (test adj3 test) or test re-test or repeat* or replic*).mp.

16 14or15

17 13 and 16

Ovid Medline; Ovid MEDLINE(R) ALL

1 exp Respiratory Tract Diseases/

2 (resp* tract diseas* or resp* diseas* or copd or chronic obstructive pulmonary diseas*

or asthma* or obstructive lung diseas* or obstructive pulmonary diseas* or cystic fibrosis or cf or (lung adj3 disease*) or healthy or healthy control).mp.

3 1 or 2

4 exp Magnetic Resonance Imaging/

5 ((magnetic resonance adj2 imag*) or MRI or magnetic resonance imag* or mr or pulmonary mri* or lung mri* or vent* magnetic resonance imag*).mp.

6 4 or 5

7 3 and 6

8 exp Xenon/ or exp Xenon Isotopes/ or exp Helium/

9 (((hyperpolarized 129Xe* or hp 129Xe* or hyperpolarized 129-Xe* or hp 129-Xe* or

hyperpolarized 129 Xe* or hp 129 Xe* or (hyperpolarized adj3 Xe*) or (hp adj3 Xe*) or Xe-129 or Xenon-129 or (hyperpolarized adj3 Xe-129) or (hyperpolarized adj3 Xenon-129) or 129Xe* or (hyperpolarized adj3 He*) or (hp adj3 He*) or 129Xe*) adj3 MRI) or (129Xe* adj3 magnetic resonance imag*) or (129-Xe* adj3 MRI) or (Xe* adj3 MRI) or (129-Xe* adj3 magnetic resonance imag*) or (Xe* adj3 magnetic resonance imaging*) or 129xe-mri or (He* adj3 MRI) or (He* adj3 magnetic resonance imag*) or (hyperpolarized adj3 gas*) or (hyperpolarized adj3 noble gas*)).mp.

10 8 or 9

11 7 and 10

12 (ventilation defect percent* or vdp or ventilation defect* or ventilation volume percent* or (ventilation adj3 percent*) or ventilated volume percent* or (ventilated adj3 percent*) or (vent* adj3 volume) or ventilation heterogeneity index or vhi or (ventilat* adj3 hetero*)).mp.

13 11 and 12

14 exp "reproducibility of results"/

15 (variab* or reproducib* or (test adj3 test) or test re-test or repeat* or replic*).mp.

16 14or15

17 13 and 16

**Supplement Table S1. GRADE Factor Definitions and Explanations for Downgrading**

| Factor | Definition | Downgrade | Explanation |
| --- | --- | --- | --- |
| Inconsistency | Heterogeneity of results and methods | 0 Acceptable | Studies use the exact same algorithmic method |
|  |  | -1 Serious | Studies use the same underlying algorithmic method |
|  |  | -2 Very Serious | Studies use a variety of multiple underlying algorithmic methods |
| Imprecision | Smaller sample sizes yield greater uncertainty and imprecision of results | 0 Acceptable | Total combined sample size: n > 100 * |
|  |  | -1 Serious | Total combined sample size: 50 < n < 100 * |
|  |  | -2 Very Serious | Total combined sample size: n < 50 * |
| Indirectness | Direct evidence consists of research directly addressing the research question as its primary aim | 0 Acceptable | Less than 25% of the studies had variability as a secondary aim |
|  |  | -1 Serious | More than 25% of the studies had variability as a secondary aim |
|  |  | -2 Very Serious | More than 50% of the studies had variability as a secondary aim |

* Definition from COSMIN Guidelines (Mokkink, et al., 2017; Prinsen, et al., 2018; Terwee, et al., 2018)

**Supplement Table S2. Table of Excluded Studies & Rationale**

| Study | | Reason for Exclusion | Rationale |
| --- | --- | --- | --- |
| (1) | Ahmed et al., 2009 | Duplicate | Duplicate data from Kirby 2011 |
| (2) | Ahmed et al., 2010 | No relevant outcome measure reported | Mean signal ventilation gradient reported |
| (3) | Alam et al., 2023 | Duplicate | Duplicate data from Munidasa 2022 |
| (4) | Alam et al., 2024 | Did not assess variability/repeatability | Assessed response to intervention |
| (5) | Bdaiwi et al., 2024 | Did not assess variability/repeatability | Compared VDP to DDI |
| (6) | de Lange et al., 2007 | No relevant summary statistic reported | Qualitative analysis by radiologists with no relevant summary statistic performed |
| (7) | de Lange et al., 2009 | No relevant outcome measure reported | Ventilation Defect Score reported |
| (8) | Diamond et al., 2023 | Did not assess variability/repeatability | Assessed VDP pre- and post- intervention |
| (9) | Du et al., 2024 | Did not assess variability/repeatability | Explored ventilation inhomogeneity |
| (10) | R. Eddy et al., 2018 | Duplicate | Duplicate data from Kirby 2012 |
| (11) | R. L. Eddy, Fernandez Elviro, et al., 2023 | Did not assess variability/repeatability | Single scan with single quantification |
| (12) | R. L. Eddy, Mummy, et al., 2023 | Did not assess variability/repeatability | Single scan defining imaging-based phenotypes |
| (13) | R. L. Eddy et al., 2024 | Did not assess variability/repeatability | Single scan defining imaging-based phenotypes |
| (14) | Garcia Delgado et al., 2024 | Did not assess variability/repeatability | Imaging compared pre- and post- intervention |
| (15) | Guo et al., 2017 | Did not assess variability/repeatability | Compared different analysis methods |
| (16) | Hall et al., 2018 | Did not assess variability/repeatability | Compared XeMRI pre and post intervention |
| (17) | Hall et al., 2019 | Did not assess variability/repeatability | Compared XeMRI pre and post intervention |
| (18) | He, Heacock, et al., 2014 | Duplicate | Duplicate data from Ebner 2017 |
| (19) | He, Kaushik, et al., 2014 | Did not assess variability/repeatability | Compared different analysis methods |
| (20) | He et al., 2015 | Did not assess variability/repeatability | Repeated imaging with different acquisition methodology |
| (21) | He et al., 2019 | Did not assess variability/repeatability | Compared different analysis methods |
| (22) | F. Horn et al., 2014 | Duplicate | Duplicate data from Marshall 2021 |
| (23) | F. C. Horn et al., 2017 | Duplicate | Duplicate data from Marshall 2021 |
| (24) | Hughes et al., 2019 | Did not assess variability/repeatability | Repeated imaging with different gas administration methodology |
| (25) | Hussain et al., 2024 | Did not assess variability/repeatability | Compared different bias-field corrections |
| (26) | Ismail et al., 2024 | Did not assess variability/repeatability | Compared disease cohort to healthy cohort |
| (27) | Kaushik et al., 2013 | No relevant summary statistic reported | No summary statistic reported |
| (28) | Kenworthy et al., 2014 | Duplicate | Duplicate data from Marshall 2021 |
| (29) | Klimes et al., 2024 | Did not assess variability/repeatability | Compared different imaging techniques |
| (30) | Koch et al., 2019 | No relevant summary statistic reported | Reported Pearson Correlation Coefficient |
| (31) | Kooner et al., 2024 | No relevant outcome measure reported | Texture analysis |
| (32) | Kruger et al., 2014 | Did not assess variability/repeatability | Compared HeMRI pre and post intervention |
| (33) | Lee et al., 2009 | Did not assess variability/repeatability | Single scan & quantification for each participant |
| (34) | Mallallah et al., 2021 | Did not assess variability/repeatability | Systematic review |
| (35) | Marshall et al., 2023 | No relevant summary statistic reported | Reported average VDP at visits 1 and 2 |
| (36) | Mozaffaripour et al., 2024 | Did not assess variability/repeatability | Compared XeMRI pre and post intervention |
| (37) | Munidasa et al., 2021 | Duplicate | Duplicate data from Munidasa 2022 |
| (38) | Munidasa et al., 2023 | Duplicate | Duplicate data from Munidasa 2022 |
| (39) | Munidasa et al., 2024 | Did not assess variability/repeatability | Compared different imaging techniques |
| (40) | Nicholson et al., 2023 | Did not assess variability/repeatability | Followed participants post-COVID-19 |
| (41) | O’Sullivan et al., 2014 | No relevant summary statistic reported | Used ANOVA |
| (42) | Ouyang et al., 2024 | Review article | Systematic review with meta-analysis |
| (43) | Parraga et al., 2007 | No relevant summary statistic reported | Compared VDP from 129Xe and 3He; Compared different image acquisitions (diffusion weighted and non-diffusion weighted); No summary statistic for variability/repeatability reported |
| (44) | G. Paulin et al., 2014 | Duplicate | Duplicate data from Kirby 2011 |
| (45) | G. A. Paulin et al., 2015 | Duplicate | Duplicate data from Kirby 2011 |
| (46) | Poranski et al., 2016 | Duplicate | Duplicate data from Zha 2019 |
| (47) | Qing et al., 2024 | Did not assess variability/repeatability | Compared imaging pre- and post- intervention |
| (48) | Radadia et al., 2024 | Did not assess variability/repeatability | Compared imaging techniques |
| (49) | Ratjen et al., 2023 | Did not assess variability/repeatability | Compared XeMRI pre and post intervention |
| (50) | D. Roach et al., 2020 | Duplicate | Duplicate data from Roach 2022 |
| (51) | D. J. Roach et al., 2022 | Did not assess variability/repeatability | Compared different analysis methods |
| (52) | Shammi et al., 2022 | Did not assess variability/repeatability | Repeated imaging with different acquisition methodology and different gases |
| (53) | Sharma et al., 2024 | Did not assess variability/repeatability | Explored machine learning model for lung defect identification |
| (54) | Shen et al., 2023 | Did not assess variability/repeatability | Compared CT and HeMRI outcomes |
| (55) | Smith, Collier, et al., 2019 | Did not assess variability/repeatability | Repeated imaging with different gases |
| (56) | Smith, Marshall, et al., 2019 | Duplicate | Duplicate data from Smith 2020 |
| (57) | N. J. Stewart et al., 2015 | Did not assess variability/repeatability | Repeated imaging with different gas dosing methodology |
| (58) | N. Stewart et al., 2016 | Duplicate | Duplicate data from Stewart 2018 |
| (59) | Streibel et al., 2024 | Did not use hyperpolarized gas | Non-HP Gas pulmonary MRI |
| (60) | Svenningsen et al., 2014 | Did not assess variability/repeatability | Assessed defects for temporal persistence and intermittence |
| (61) | Tanabe et al., 2024 | Review article | Review article |
| (62) | Tcherner et al., 2024 | Did not assess variability/repeatability | Compared CT and XeMRI outcomes |
| (63) | Thomen et al., 2015 | Did not assess variability/repeatability | Compared HeMRI pre and post intervention |
| (64) | Virgincar et al., 2012 | Did not assess variability/repeatability | Compared different analysis methods |
| (65) | Walkup et al., 2021 | Duplicate | Duplicate data from Walkup 2024 |
| (66) | Wee et al., 2022 | Feasibility Study | Single participant feasibility study |
| (67) | West et al., 2023 | Did not assess variability/repeatability | Repeated imaging pre- and post-intervention |
| (68) | Wild et al., 2024 | Review article | Review of XeMRI for long-COVID |
| (69) | Willmering et al., 2024 | Did not assess variability/repeatability | No repeat imaging or quantification |

**References**

1. Ahmed H, Wheatley AR, Paterson NA, Parraga G: Hyperpolarized Helium-3 Magnetic Resonance Imaging Precision in Cystic Fibrosis. *Am J Respir Crit Care Med* 2009(Meeting Abstracts):A1444-.

2. Ahmed H, Choy S, Wheatley A, Etemad-Rezai R, Paterson N, Parraga G: Mapping and quantifying temporal dynamics of ventilation heterogeneity in cystic fibrosis using hyperpolarized helium-3 magnetic resonance imaging: Developing new imaging measurements for clinical trials. *Am J Respir Crit Care Med* 2010; 181(1 MeetingAbstracts).

3. Alam FS, Zanette B, Munidasa S, et al.: Intra- and Inter-visit Repeatability of 129Xenon Multiple-Breath Washout MRI in Children With Stable Cystic Fibrosis Lung Disease. *J Magn Reson Imaging* 2023; 58:936–948.

4. Alam FS, Munidasa S, Zanette B, et al.: Assessing 129Xe multi-breath washout MRI response to elexacaftor/tezacaftor/ivacaftor intervention in pediatric CF. *J Cyst Fibros* 2024.

5. Bdaiwi A, Willmering MM, Walkup L, Woods JC, Cleveland ZI: Quantifying Spatial Distribution of Ventilation Defects in Lung Imaging With Hyperpolarized 129Xenon MRI. *Am J Respir Crit Care Med* 2024; 209((Bdaiwi, Willmering, Walkup, Woods, Cleveland) Division of Pulmonary Medicine, Cincinnati Children’s Hospital Medical Center, Cincinnati, OH, United States).

6. de Lange EE, Altes TA, Patrie JT, et al.: The variability of regional airflow obstruction within the lungs of patients with asthma: Assessment with hyperpolarized helium-3 magnetic resonance imaging. *Journal of Allergy and Clinical Immunology* 2007; 119:1072–1078.

7. de Lange EE, Altes TA, Patrie JT, et al.: Changes in regional airflow obstruction over time in the lungs of patients with asthma: Evaluation with3He MR imaging. *Radiology* 2009; 250:567–575.

8. Diamond V, Ha B, Eddy R, Rayment J: Hyperpolarized 129Xe pulmonary MRI and multiple-breath washout to measure response to elexacaftor-tezacaftor-ivacaftor therapy in children. *Journal of Cystic Fibrosis* 2023; 22(Supplement 3):S57–S58.

9. Du K, Lu J, Mummy D, Driehuys B, Huang YT: 129xe MRI With Optimized Bias Field Correction to Assess Ventilation Gradients in Individuals With Normal Lung Function. *Am J Respir Crit Care Med* 2024; 209((Du) Biomedical Engineering, Duke University, Durham, NC, United States(Lu) Medical Physics Graduate Program, Duke University, Durham, NC, United States(Mummy, Driehuys) Department of Radiology, Duke University Medical Center, Duke University, Durham, NC,).

10. Eddy R, Svenningsen SL, McCormack DG, Parraga G: Determining the minimal clinically important difference: MRI ventilation defect percent in asthmatics. *Am J Respir Crit Care Med* 2018; 197(MeetingAbstracts).

11. Eddy RL, Fernandez Elviro C, Diamond VM, Baird R, Rayment JH: Lung Structure-function in Pediatric Survivors of Congenital Diaphragmatic Hernia. *Am J Respir Crit Care Med* 2023; 207.

12. Eddy RL, Mummy D, Dai H, et al.: 129Xe Magnetic Resonance Imaging-based Phenotypes of Long COVID: A Multi-center Evaluation. *Am J Respir Crit Care Med* 2023; 207.

13. Eddy RL, Mummy D, Zhang S, et al.: Cluster analysis to identify long COVID phenotypes using 129Xe magnetic resonance imaging: a multicentre evaluation. *European Respiratory Journal* 2024; 63:2302301.

14. Garcia Delgado GM, Shammi U, Ruppel M, et al.: Assessment of Gas Exchange With Hyperpolarized 129Xe MR Imaging in Mild Asthmatics When Administered a Vasodilator. *Am J Respir Crit Care Med* 2024; 209((Garcia Delgado, Ruppel) Chemical and Biomedical Engineering, University of Missouri, Columbia, MO, United States(Shammi) NextGen Precision Health Institute, Columbia, MO, United States(Mummy) Duke University Medical Center, Durham, NC, United States(Lu)).

15. Guo F, Svenningsen S, Kirby M, et al.: Thoracic CT-MRI coregistration for regional pulmonary structure-function measurements of obstructive lung disease. *Med Phys* 2017; 44:1718–1733.

16. Hall C, Quirk JD, Goss C, et al.: Regional ventilation changes in severe asthma after bronchial thermoplasty. *Am J Respir Crit Care Med* 2018; 197(MeetingAbstracts).

17. Hall C, Quirk J, Goss C, et al.: Bronchial thermoplasty effect on ventilation guided by hyperpolarized 129Xe MRI in severe asthma. 2019; 54.

18. He M, Heacock T, Kaushik SS, et al.: Hyperpolarized 129Xe MRI to quantify regional ventilation differences in older versus younger asthmatics. *Am J Respir Crit Care Med* 2014; 189(MeetingAbstracts).

19. He M, Kaushik SS, Robertson SH, et al.: Extending semiautomatic ventilation defect analysis for hyperpolarized 129Xe ventilation MRI. *Acad Radiol* 2014; 21:1530–1541.

20. He M, Robertson SH, Kaushik SS, et al.: Dose and pulse sequence considerations for hyperpolarized 129Xe ventilation MRI. *Magn Reson Imaging* 2015; 33:877–885.

21. He M, Zha W, Tan F, Rankine L, Fain S, Driehuys B: A Comparison of Two Hyperpolarized 129Xe MRI Ventilation Quantification Pipelines: The Effect of Signal to Noise Ratio. *Acad Radiol* 2019; 26:949–959.

22. Horn F, Marshall H, Barber D, et al.: Quantitative mapping of treatment response in the lungs of asthmatics using hyperpolarized ventilation helium-3 MRI. *Am J Respir Crit Care Med* 2014; 189(MeetingAbstracts).

23. Horn FC, Marshall H, Collier GJ, et al.: Regional ventilation changes in the lung: Treatment response mapping by using hyperpolarized gas MR imaging as a quantitative biomarker. *Radiology* 2017; 284:854–861.

24. Hughes PJC, Smith L, H.-F. C, et al.: Assessment of the influence of lung inflation state on the quantitative parameters derived from hyperpolarized gas lung ventilation MRI in healthy volunteers. *J Appl Physiol (1985)* 2019; 126:183–192.

25. Hussain R, Plummer JW, Bdaiwi AS, Willmering MM, Walkup LL, Cleveland ZI: Improved Quantification of Ventilation Heterogeneity in Hyperpolarized Xenon MRI Using Physics-rooted Bias-field Correction. *Am J Respir Crit Care Med* 2024; 209((Hussain, Plummer, Bdaiwi, Willmering) Center for Pulmonary Imaging Research (CPIR), Cincinnati Children’s Hospital Medical Center, Cincinnati, OH, United States(Walkup, Cleveland) Pulmonary Medicine, Cincinnati Children’s Hospital Medical Center, Cincinn).

26. Ismail B, Plummer JW, Willmering M, et al.: Pulmonary Gas-exchange Abnormalities Prior to Hematopoietic Stem-cell Transplantation Detected With Xenon MRI: Preliminary Results From the Transpire Study. *Am J Respir Crit Care Med* 2024; 209((Ismail) Center for Pulmonary Imaging Research, Division of Pulmonary Medicine, Cincinnati Children’s Medical Center, Cincinnati, OH, United States(Plummer) Pulmonary Medicine, Cincinnati Children’s Hospital Medical Center, Cincinnati, OH, United States(W).

27. Kaushik SS, Liljeroth M, Virgincar RS, et al.: Reproducibility of hyperpolarized 129xe MRI: Alveolar capillary gas-transfer spectroscopy and imaging. *Am J Respir Crit Care Med* 2013; 187(MeetingAbstracts).

28. Kenworthy JC, Marshall H, Parra-Robles J, et al.: Reproducibility of lung ventilation volume from helium-3 and proton MRI in asthmatics. *Am J Respir Crit Care Med* 2014; 189(MeetingAbstracts).

29. Klimes F, Kern AL, Voskrebenzev A, et al.: Free-breathing 3D phase-resolved functional lung MRI vs breath-hold hyperpolarized 129Xe ventilation MRI in patients with chronic obstructive pulmonary disease and healthy volunteers. *Eur Radiol* 2024.

30. Koch MF, Mugler JP, Mata JF, et al.: Comparison of hyperpolarized 3He and 129Xe MR imaging in patients with cystic fibrosis. *Am J Respir Crit Care Med* 2019; 199.

31. Kooner HK, Sharma M, McIntosh MJ, et al.: 129Xe MRI Ventilation Textures and Longitudinal Quality-of-Life Improvements in Long-COVID. *Acad Radiol* 2024; 31:3825–3836.

32. Kruger SJ, Niles DJ, Dardzinski B, et al.: Hyperpolarized Helium-3 MRI of exercise-induced bronchoconstriction during challenge and therapy. *Journal of Magnetic Resonance Imaging* 2014; 39:1230–1237.

33. Lee EY, Sun Y, Zurakowski D, Hatabu H, Khatwa U, Albert MS: Hyperpolarized 3He MR imaging of the lung: Normal range of ventilation defects and PFT correlation in young adults. *J Thorac Imaging* 2009; 24:110–114.

34. Mallallah F, Packham A, Lee E, Hind D: Is hyperpolarised gas magnetic resonance imaging a valid and reliable tool to detect lung health in cystic fibrosis patients? a cosmin systematic review. *Journal of Cystic Fibrosis* 2021; 20:906–919.

35. Marshall H, Smith LJ, Biancardi A, et al.: 129XE MRI PHENOTYPING AND LONGITUDINAL CHANGE IN PATIENTS WITH ASTHMA AND/OR COPD AND NORMAL PULMONARY FUNCTION TESTS. *Thorax* 2023; 78(Supplement 4):A215-.

36. Mozaffaripour A, Tcherner S, Sharma M, et al.: 129Xe MRI and Oscillometry Evidence of Improved Small Airways Dysfunction After 6-weeks ICS/LABA/LAMA Therapy in Moderate Asthma. *Am J Respir Crit Care Med* 2024; 209((Mozaffaripour) School of Biomedical Engineering, Western University, London, ON, Canada(Tcherner, Sharma, Kooner, Parraga) Department of Medical Biophysics, Western University, London, ON, Canada(Mcintosh) Department of Radiology, University of Iowa, Iow).

37. Munidasa S, Seethamraju R, Au J, et al.: Inter-visit reproducibility of free-breathing lung magnetic resonance imaging in cystic fibrosis. *J Cyst Fibros* 2021; 20(Supplement 2):S80-.

38. Munidasa S, Zanette B, Couch M, et al.: Inter- and intravisit repeatability of free-breathing MRI in pediatric cystic fibrosis lung disease. *Magn Reson Med* 2023; 89:2048–2061.

39. Munidasa S, Zanette B, M.-P. D, et al.: Comparison of 3D UTE free-breathing lung MRI with hyperpolarized 129Xe MRI in pediatric cystic fibrosis. *Magn Reson Med* 2024((Munidasa, Zanette, Braganza, Li, Ratjen, Santyr) Translational Medicine Program, The Hospital for Sick Children, Toronto, ON, Canada(Munidasa, Santyr) Department of Medical Biophysics, University of Toronto, Toronto, ON, Canada(Dumas, Wee, Ratjen) Divisi).

40. Nicholson JM, Matheson AM, Kooner HK, Mcintosh MJ, Svenningsen S, Parraga G: Unique MRI Phenotypes Help Explain Post-Acute COVID-19 Syndrome. *Am J Respir Crit Care Med* 2023; 207.

41. O’Sullivan B, Couch M, Roche JP, et al.: Assessment of repeatability of hyperpolarized gas MR ventilation functional imaging in cystic fibrosis. *Acad Radiol* 2014; 21:1524–1529.

42. Ouyang T, Tang Y, Zhang C, Yang Q: Phase-resolved MRI for measurement of pulmonary perfusion and ventilation defects in comparison with dynamic contrast-enhanced MRI and 129Xe MRI. *BMJ Open Respir Res* 2024; 11.

43. Parraga G, Ouriadov A, Evans A, et al.: Hyperpolarized 3He ventilation defects and apparent diffusion coefficients in chronic obstructive pulmonary disease: Preliminary results at 3.0 Tesla. *Invest Radiol* 2007; 42:384–391.

44. Paulin G, Svenningsen S, Mohan S, et al.: Longitudinal hyperpolarized 3he magnetic resonance imaging of adult cystic fibrosis: Pilot study results. *Am J Respir Crit Care Med* 2014; 189(MeetingAbstracts).

45. Paulin GA, Svenningsen S, Jobse BN, et al.: Differences in hyperpolarized 3He ventilation imaging after 4 years in adults with cystic fibrosis. *Journal of Magnetic Resonance Imaging* 2015; 41:1701–1707.

46. Poranski M, Zha W, Cadman R, et al.: Ventilation and perfusion MRI of cystic fibrosis. 2016; 51:354.

47. Qing K, Altes TA, Mugler JP 3rd, et al.: Pulmonary MRI with hyperpolarized xenon-129 demonstrates novel alterations in gas transfer across the air-blood barrier in asthma. *Med Phys* 2024; 51:2413–2423.

48. Radadia N, Priel E, Friedlander Y, et al.: Ventilation Defect Burden Quantified by Technegas SPECT and 129Xe MRI Predicts Lung Cancer Resection Outcomes. *Am J Respir Crit Care Med* 2024; 209((Radadia, Jamal, Nair, Svenningsen) Department of Medicine, McMaster University, Hamilton, ON, Canada(Priel, Huang) Firestone Institute for Respiratory Health, St. Joseph’s Healthcare Hamilton, Hamilton, ON, Canada(Friedlander, Konyer) Imaging Research Ce).

49. Ratjen F, Stanojevic S, Walkup L, et al.: Response in imaging and lung function outcomes in the HyPOINT study. *Journal of Cystic Fibrosis* 2023; 22(Supplement 3):S68–S69.

50. Roach D, Willmering M, Plummer J, et al.: Hyperpolarized 129Xenon MRI Ventilation Defect Quantification Pipelines Across Multiple Pulmonary Diseases. *D110 NEW INSIGHTS FROM ADVANCED IMAGING* 2020:A7893–A7893.

51. Roach DJ, Willmering MM, Plummer JW, et al.: Hyperpolarized 129Xenon MRI Ventilation Defect Quantification via Thresholding and Linear Binning in Multiple Pulmonary Diseases. *Acad Radiol* 2022; 29:S145–S155.

52. Shammi UA, D’Alessandro MF, Altes T, et al.: Comparison of Hyperpolarized 3He and 129Xe MR Imaging in Cystic Fibrosis Patients. *Acad Radiol* 2022; 29:S82–S90.

53. Sharma M, Kirby M, McCormack DG, Parraga G: Machine Learning and CT Texture Features in Ex-smokers with no CT Evidence of Emphysema and Mildly Abnormal Diffusing Capacity. *Acad Radiol* 2024; 31:2567–2578.

54. Shen W, Zhang X, Angelini ED, et al.: Lung Microstructure and Regional Function of Quantitative Emphysema Subtypes on Coregistered Computed Tomography and Hyperpolarized Gas Magnetic Resonance Imaging Scans: The M E S A - C O P D Study. *Am J Respir Crit Care Med* 2023; 207.

55. Smith L, Collier G, Marshall H, et al.: A comparison of ventilation MRI using hyperpolarised 3He and 129Xe to assess cystic fibrosis lung disease. *Journal of Cystic Fibrosis* 2019; 18(Supplement 1):S117-.

56. Smith L, Marshall H, Norquay G, et al.: 129Xe ventilation MRI and LCI to assess acute maximal exercise as a method of airway clearance. *Journal of Cystic Fibrosis* 2019; 18(Supplement 1):S116–S117.

57. Stewart NJ, Norquay G, Griffiths PD, Wild JM: Feasibility of human lung ventilation imaging using highly polarized naturally abundant xenon and optimized three-dimensional steady-state free precession. *Magn Reson Med* 2015; 74:346–352.

58. Stewart N, H.-F. C, Hughes P, et al.: Comparison of 3He and 125Xe MRI for evaluation of lung microstructure and ventilation at 1.5 T. *European Respiratory Journal* 2016; 48(Supplement 60).

59. Streibel C, Willers CC, Bauman G, et al.: Long-term pulmonary outcome of children with congenital diaphragmatic hernia: functional lung MRI using matrix-pencil decomposition enables side-specific assessment of lung function. *Eur Radiol* 2024; 34:3773–3785.

60. Svenningsen S, Guo F, Kirby M, et al.: Pulmonary functional magnetic resonance imaging: asthma temporal-spatial maps. *Acad Radiol* 2014; 21:1402–1410.

61. Tanabe N, Nakagawa H, Sakao S, et al.: Lung imaging in COPD and asthma. *Respir Investig* 2024; 62:995–1005.

62. Tcherner S, Mozaffaripour A, Kooner HK, et al.: CT Mucus Score Predicts Response to 6-weeks ICS/LABA/LAMA in Moderate Asthma. *Am J Respir Crit Care Med* 2024; 209((Tcherner, Kooner, Sharma, Aseffa, Parraga) Department of Medical Biophysics, Western University, London, ON, Canada(Mozaffaripour) School of Biomedical Engineering, Western University, London, ON, Canada(Mcintosh) Department of Radiology, University of I).

63. Thomen RP, Sheshadri A, Quirk JD, et al.: Regional Ventilation Changes in Severe Asthma after Bronchial Thermoplasty with 3He MR Imaging and CT. *Radiology* 2015; 274:250–259.

64. Virgincar RS, Kaushik SS, Cleveland ZI, et al.: Hyperpolarized 129Xe ventilation imaging in healthy subjects and subjects with COPD: Correlating reader based scoring with automatic segmentation. *Am J Respir Crit Care Med* 2012; 185(MeetingAbstracts).

65. Walkup L, Roach D, Santyr G, et al.: 129Xe MRI is a repeatable measure of regional ventilation in children with stable CF. *Journal of Cystic Fibrosis* 2021; 20(Supplement 2):S253–S254.

66. Wee WB, Zanette B, Munidasa S, et al.: Feasibility and Reliability of Hyperpolarized 129Xe MRI in Pediatric Primary Ciliary Dyskinesia. *Am J Respir Crit Care Med* 2022; 205.

67. West ME, Spielberg DR, Roach DJ, et al.: Short-term structural and functional changes after airway clearance therapy in cystic fibrosis. *Journal of Cystic Fibrosis* 2023; 22:926–932.

68. Wild JM, Gleeson F V, Svenningsen S, et al.: Review of Hyperpolarized Pulmonary Functional 129 Xe MR for Long-COVID. *J Magn Reson Imaging* 2024; 59:1120–1134.

69. Willmering M, Hysinger E, Janjindamai C, et al.: Assessment of Obstructive and Restrictive Lung Disease Via MRI in Bronchopulmonary Dysplasia Patients. *Am J Respir Crit Care Med* 2024; 209((Willmering, Hysinger, Janjindamai, Soderlund, Hussain, Plummer, Bdaiwi, Critser, Parikh, Amin, Woods) Cincinnati Children’s Hospital Medical Center, Cincinnati, OH, United States).
